# Effectiveness of Digital Mental Health Interventions for Children and Adolescents

**DOI:** 10.3390/children12030353

**Published:** 2025-03-12

**Authors:** José María Fernández-Batanero, José Fernández-Cerero, Marta Montenegro-Rueda, Daniel Fernández-Cerero

**Affiliations:** 1 Department of Teaching and Educational Organization, University of Seville, 41013 Sevilla, Spain; jfcerero@us.es; 2 Department of Didactics and School Organisation, University of Granada, 52005 Melilla, Spain; mmontenegro@ugr.es; 3 Department of Stomatology, University of Seville, 41009 Sevilla, Spain; dfcerero@us.es

**Keywords:** mental health, digital technology, digital intervention, children, adolescents, emotional well-being

## Abstract

Background/Objectives: Children’s mental health is an issue of growing global concern, with a significant impact on children’s emotional, social and cognitive development. In recent years, digital apps and platforms have emerged as innovative tools to address mental health challenges. This systematic review aims to evaluate the effectiveness of these technologies in supporting children’s and adolescents’ mental health. Methods: A systematic search of PubMed, PsycINFO, Scopus and Web of Science databases was conducted. Results: The results suggest that digital apps and platforms have significant potential to support children’s mental health. However, their effectiveness depends on factors such as app design, parental involvement and cultural adaptation. Conclusions: The inclusion of gamified elements and integration with traditional mental health strategies can enhance outcomes.

## 1. Introduction

Child mental health is a global priority in the current context, marked by increasing challenges related to accelerated social change and technological pressures [1]. It is estimated that one in five children and adolescents worldwide experience a mental health disorder, posing a considerable challenge to families, communities and health systems [2]. These problems, exacerbated by factors such as unequal access to health resources, the stigma associated with psychological disorders, and natural disasters such as the COVID-19 pandemic, have led to a significant increase in the incidence of anxiety, depression and related mental health disorders in children and adolescents [3]. In this context, technologies emerge as promising tools to address children’s mental health needs, but their implementation and effectiveness still raise crucial questions that require in-depth and critical analysis.

The accelerating advance in digital technologies, including mobile apps, online platforms and artificial intelligence-based solutions, has transformed the healthcare landscape, offering new opportunities for child mental health assessment, intervention and monitoring [4]. Recent studies indicate that these technologies can improve access to mental health services, especially in regions where resources are limited [5]. On the other hand, digital resources play a key role in mental health prevention and education by providing accessible information for both children and their families. Through these digital tools, it is possible to promote knowledge about emotional well-being and teach effective coping strategies in various situations [6]. However, significant challenges remain, such as the lack of rigorous scientific validation of many commercially available apps, ethical concerns related to data privacy, and unequal access due to digital divides [7]. In this sense, while technology brings multiple benefits in the field of mental health, its implementation should not be considered an absolute substitute for professional intervention. It is essential that these digital tools are integrated with traditional services, enabling more comprehensive and effective care for patients, especially in cases that require a specialised clinical approach [8].

Despite the increasing proliferation of technological interventions, evidence on their effectiveness and long-term sustainability remains fragmented and, in many cases, limited [9]. For example, while some studies highlight the positive impact of technology-based interventions on reducing symptoms of anxiety and depression [10], others point to a lack of standardisation in the design and evaluation of such tools, making it difficult to compare and generalise results [11]. This disparity of results underscores the urgent need to consolidate existing knowledge across the literature to identify trends, gaps and priority areas for future research. These gaps not only hinder theoretical advancement but also compromise the effectiveness and equity of technological interventions in clinical practice.

A review of the literature in this field is therefore necessary to address these challenges and contribute to the development of a more robust conceptual and empirical framework. Thus, this study seeks to critically synthesise the available evidence on the effectiveness of technologies in supporting children’s mental health, highlighting both their strengths and limitations. Through a comprehensive analysis of recent studies, it aims to identify consistent patterns, highlight the most promising innovations, and propose priority areas for future research. This approach will not only clarify the state of the art, but also inform decision-making by researchers, health specialists and policy makers. The review is framed in a historical moment of growing interest in the intersection between technology and mental health, which makes it particularly relevant and timely. The review is framed in a historical moment of growing interest in the intersection between technology and mental health, which makes it particularly relevant and timely. We have conducted a systematic review study that aimed to evaluate the effectiveness of apps and digital platforms as interventions to improve the mental health of children and adolescents. Although this is a well-studied topic, it is a valuable update, addressing the specific gap in this particularly rapidly developing field of digital health apps, adding studies with more up-to-date technologies.

This research aligns with contemporary debates in psychology, psychiatry and technology, including the role of interdisciplinary approaches in the design of digital interventions [12]. For example, the integration of user-centred design principles and participatory research methodologies has been highlighted as a key strategy for improving the uptake and effectiveness of mental health technologies [13]. Furthermore, the ‘digital psychology’ framework provides a conceptual basis for understanding how human interactions with digital technologies can influence mental health and well-being [14].

On the other hand, the effectiveness that technologies can have, not only in clinical settings, but also in educational settings, has been demonstrated [15]. Therefore, the use of video games and gamification strategies has proven to be an effective alternative to facilitate access and improve continuity in psychological treatments for children and adolescents. In this sense, initiatives such as ‘Harry’s PathwaysToCare’ have been created with the purpose of guiding young people in the process of seeking and using mental health services [16]. However, despite their advantages, the implementation of these technologies in the professional mental health setting remains limited. This is largely due to a lack of understanding of how they work and concerns related to the security and privacy of user data [17].

Following this line, the present study pursues the following objectives:

(1) To evaluate the effectiveness of various technologies on mental health in children and adolescents;
(2) To identify the advantages and limitations of these technologies for psychological well-being;
(3) To propose a framework for future research on the implementation of technologies for child and adolescent mental health.

To this end, the study answers the following research questions:

RQ1: What evidence exists on the effectiveness of digital technologies on the mental health of children and adolescents?

RQ2: What are the main challenges and barriers in the use of digital technologies for psychological well-being?

RQ3: What guidelines can guide future research on the implementation of mental health technologies for children and young people?

## 2. Methods

### 2.1. Review Methodology

This systematic review was conducted in accordance with the Preferred Reporting Items for Systematic Reviews and Meta-Analyses (PRISMA) guidelines [18] (Supplementary Materials S1). The implementation of these guidelines ensures a clear and comprehensive methodology, promoting transparency and rigour in the study selection process for systematic reviews. A search was conducted to identify studies exploring the efficacy of technologies on child and adolescent mental health.

### 2.2. Formulation of the Research Question

The search query was conducted using the PICO model [19] which includes four core elements: person or problem (P), indicator intervention (I), comparison (c) and outcome (O). The following aspects were considered: P—child or adolescent students; I—mental health; C—digital intervention or tool and O—impact of digital tools on mental health.

### 2.3. Search Strategy

The search was conducted in recognised academic databases, including PubMed, Scopus, Web of Science and PsycINFO, to identify studies published between 2020 and 2024. The delimitation of the study period to the years 2020–2024 was in response to several factors. Firstly, the COVID-19 pandemic represented a turning point in the development and implementation of digital mental health interventions, accelerating the adoption of technologies and generating a significant increase in the research and application of these strategies. The purpose of the study is to analyse effectiveness in a scenario characterised by the increasing digitisation of mental health care, largely driven by the conditions imposed since 2020 following the pandemic.

Keywords related to mental health, digital technology, digital interventions, children, adolescents and emotional well-being were used. Boolean operators (AND, OR) were applied to optimise the retrieval of relevant studies. In addition, reference lists of selected studies were reviewed to identify additional relevant publications.

### 2.4. Inclusion and Exclusion Criteria

Based on the inclusion and exclusion criteria, a number of guidelines were established to ensure that the selected studies were relevant and met the objectives of the review.

The inclusion criteria were as follows:

- Published between 2020 to 2024;
- Publications in scientific articles;
- Publications in English;
- Related to the field of study.

The exclusion criteria were as follows:

- Published outside the established time interval;
- Published in a format other than a scientific article (congress, thesis, etc.);
- Published in a language other than English;
- Research not related to the field of study.

### 2.5. Selection of Studies and Selection Process

Study selection was performed in several stages. First, the search results were combined using the desktop reference manager Mendeley and duplicate studies were removed. Second, two researchers independently screened the titles and abstracts of articles identified in the initial search to discard studies that did not meet the inclusion criteria. Subsequently, a full-text review of the pre-selected studies was conducted to confirm relevance and data extraction.

**Figure 1:**
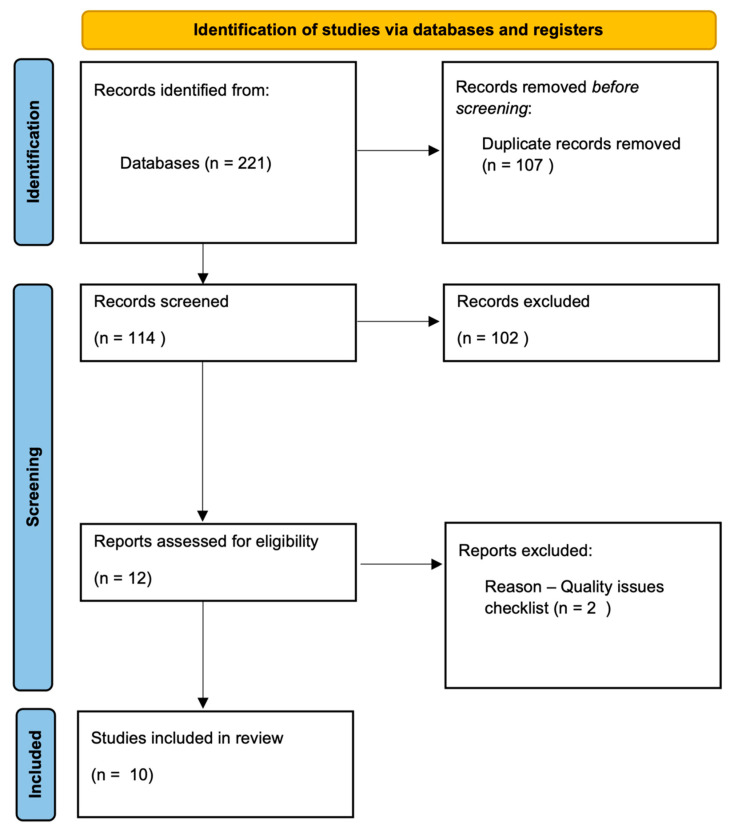
Flow chart.

To ensure the reliability of the process, the inter-rater agreement index was calculated using Cohen’s Kappa coefficient, yielding a value of 0.95, indicating a high level of inter-rater agreement. Any discrepancies were resolved by discussion between the researchers or, if necessary, with the intervention of a third evaluator. The results can be visualised in Figure 1.

### 2.6. Methodological Quality Assessment

Two reviewers independently determined the methodological quality of the selected studies using the critical appraisal tools for non-randomised studies of the Joanna Briggs Institute (JBI) at the University of Adelaide (Australia) [20]. This tool is designed to determine the robustness and validity of research, as well as to assess the methodological quality of a study and to determine the extent to which a study has excluded or minimised the possibility of bias in its design, conduct and/or analysis. The version for quantitative studies was adapted, with the cut-off point being 4 for acceptance for inclusion in this review (Supplementary Materials S2) [20].

To assess the risk of bias in the non-randomised studies included in this review, the Risk of Bias in Non-randomised Studies of Interventions (ROBINS-I) tool recommended by Cochrane was used. This tool allows us to identify and classify bias in seven key domains: confounders, participant selection, intervention classification, deviations from the intended intervention, missing data, outcome measurement and selection of reported outcomes. Each domain was assessed with a risk rating of low (green), moderate (yellow), high (orange) or critical (red) bias, providing a comprehensive assessment of the overall risk of each study. The application of this tool ensures greater transparency and methodological rigour in the synthesis of findings, allowing the robustness of the evidence analysed to be contextualised (Supplementary Materials S3) [21].

### 2.7. Data Extraction and Data Analysis

The following information was extracted from the included studies: authors, date of publication, methodological design, population size and characteristics, type of digital intervention implemented, country of implementation and main findings. To ensure consistency in data extraction, a standardised template incorporating the variables mentioned above was used.

Data analysis was conducted at two levels: descriptive and thematic. First, a quantitative analysis was conducted to examine the distribution of studies in terms of frequency of publication by year, types of digital interventions and mental health conditions addressed. Secondly, a qualitative analysis was conducted to identify patterns in the effectiveness of digital interventions. Study results were categorised according to reported effects on child and adolescent mental health. To do this, a response categorisation process was applied, where quantitative data were transformed into qualitative categories to facilitate analysis and interpretation. Segmentation and clustering techniques were used to identify patterns and groupings within the numerical responses. SPSS statistical software (v.29 for MacOS) was used to group results into clusters with significant similarities, improving understanding of key trends in digital mental health interventions.

## 3. Results

A search of four databases was conducted, resulting in 211 records. Of these, 10 articles were included in the review (Table 1). Finally, in order to extract relevant data to help answer our research questions, the selected studies were analysed and their data extracted using a standardised template that included information on methodological design, population, digital intervention and main findings.

The number of published studies on digital mental health fluctuated between 2020 and 2024, reflecting the impact of the global pandemic on interest and development in this topic. In 2020, only one study was identified, which is consistent with the time required to design and implement research after the onset of the health crisis. In 2021, the number of publications increased to four, suggesting a growth in attention to and exploration of digital mental health interventions. Subsequently, in 2023 and 2024, the number of studies decreased to two, which could indicate a stabilisation phase in scientific output. This pattern highlights how research responded to emerging needs driven by the pandemic and how, in recent years, the publication of studies appears to have reached a point of normalisation (Supplementary Materials S3).

Looking at the geographical origin of the studies collected, a large percentage of the articles come from South and Southeast Asia, with countries such as India, Indonesia and Bangladesh showing growth and interest in dealing with technologies and the mental health of users. On the other hand, studies were also found from countries from other continents such as Europe (Norway), South America (Chile), Africa (Kenya and Egypt) and North America (Canada and the United States). This global picture not only shows the universality of interest in digital mental health but also suggests that innovative solutions developed in these diverse contexts can be shared and adapted globally.

**Figure 2:**
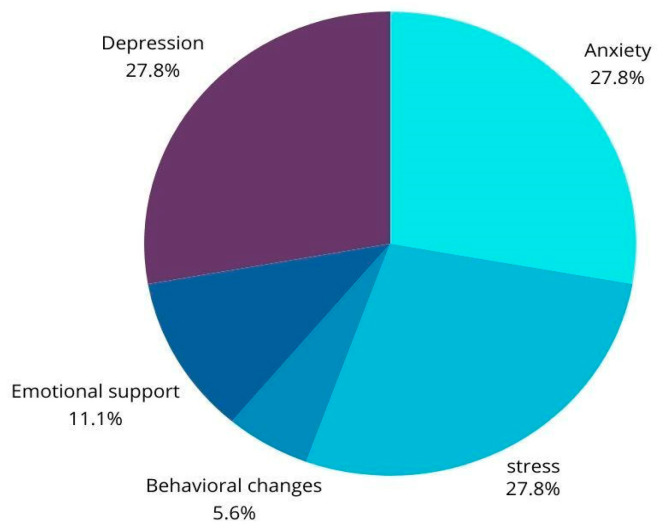
Distribution according to study topics.

Figure 2 illustrates the distribution of study topics in digital mental health interventions, focusing on five main categories, each of which represents a significant area of research within the field of mental health. Depression, anxiety and stress each occupy 27.8% of the total number of studies, reflecting the high priority that these areas represent in current research. This balance shows that researchers are devoting similar resources to explore how digital interventions can help manage these disorders, which are prevalent and have a considerable impact on the world’s population. On the other hand, we find emotional support (11.1%) and behavioural changes (5.6%). These studies address how digital interventions can influence behaviour modification, an essential aspect of improving overall well-being and managing mental health conditions.

Addressing the main findings of the collected studies, the results have been categorised into five main areas:

- Reduction in Anxiety and Stress: This section refers to digital interventions designed to help reduce anxiety and stress levels in young people. They often include relaxation techniques, cognitive behavioural therapy, and guided activities aimed at relieving emotional and psychological tension.
- Increase in Mental Health Awareness and Management: Focuses on increasing mental health awareness among young people and their caregivers. This may include education about the symptoms of different psychological disorders, strategies for managing them, and the importance of seeking professional help when necessary. Apps and platforms often provide informative resources and self-assessments.
- Active Participation in Mental Health: This outcome highlights the importance of active and engaged participation of users in their own mental health process. Interventions may include interactive modules, emotion diaries, and discussion forums that allow users to actively engage in their wellness journey.
- Reduction in Depressive Symptoms: This section addresses specific interventions to reduce symptoms associated with depression, such as persistent sadness, lack of interest in daily activities, and concentration problems. These interventions are often guided by psychological therapy principles and supported by regular follow-up.
- Improvements in General Well-being: Refers to interventions that aim to improve the general well-being of individuals, focusing not only on clinical aspects, but also on improving quality of life. This may include promoting healthy habits, developing social skills and strengthening emotional resilience

Based on the results obtained after analysing the main findings, it stands out that interventions focused on anxiety and stress reduction have shown a high effectiveness, reaching 87%. These interventions usually include programmes that implement stress management techniques and mindfulness, proving to be effective tools to help young people control their anxiety levels. On the other hand, interventions that promote increased knowledge and management of mental health show an effectiveness of 85%, focusing their action on educating young people about mental health problems and strategies for their management, thus increasing their awareness and skills to manage their own mental health.

**Figure 3:**
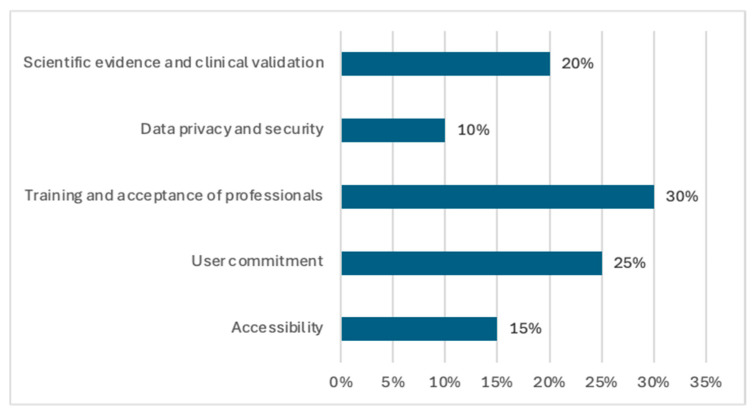
Challenges of implementing technologies in mental health interventions.

Active participation in mental health, which includes interactive tools and progress monitoring, has proven to be especially effective with 89% effectiveness, encouraging greater engagement of youth in their treatment. The category with the highest reported effectiveness, at 90%, is the reduction in depressive symptoms. These interventions have shown positive results in helping adolescents regain interest in daily activities and improve their overall mood. Finally, interventions aimed at improvements in general well-being, ranging from promoting healthy lifestyles to developing social skills and emotional resilience, have shown 86% effectiveness. These programmes seek not only to reduce the symptoms of specific disorders but also to promote a comprehensive improvement in the quality of life of young people. In this sense, digital interventions in child and adolescent mental health are effective tools on a variety of fronts, offering significant support in the management and improvement of mental health. However, the findings reveal challenges for its implementation in both clinical and educational contexts (Figure 3). The figure shows the percentage distribution of the main barriers to the implementation of technologies in the health of young people. It can be seen that the training and acceptance of professionals represents the greatest limitation (30%), suggesting the need for training strategies to improve the adoption of these technologies in the clinical setting. This is followed by user commitment (25%) and scientific evidence and clinical validation (20%), indicating that adherence of young people and the lack of robust studies on the efficacy of these technologies remain major challenges. To a lesser extent, accessibility (15%) and data privacy and security (10%) also constitute barriers, although with less relative impact compared to the previous factors. These findings highlight the importance of addressing both professional training and scientific evidence to foster effective integration of technologies in youth health, as well as the importance of continuing to develop and implement technologies tailored to the mental health needs of this population.

## 4. Discussion

The following are the discussions organised according to the research questions posed above:

RQ1: What evidence exists on the effectiveness of digital technologies in intervening and supporting child and adolescent mental health?

The study of previous research has confirmed the positive effect of the use of digital tools for the improvement of children and adolescent mental health. The literature review has shown that their application generates significant benefits in several areas. On the one hand, the efficacy of digital interventions for childhood anxiety, encompassing mobile applications, virtual reality and online therapies, has been the subject of study. The results indicate that these tools can facilitate access and standardisation of treatment, although further ethical and practical regulation is still required [32]. Following this line, other studies conducted research in which they evaluated the effectiveness of a digital platform designed for the treatment of anxiety and depression in adolescents. Through various methodologies and digital tools, they analysed the evolution of participants who used the platform as part of their mental health intervention. The results showed a significant improvement in their anxiety symptoms, suggesting that digital interventions can be an effective tool to expand access to psychological treatments in this population [33], as well as school-based interventions to reduce anxiety in children and adolescents that generate small but significant benefits, although their long-term impact remains limited [34]. However, it has also been reflected that it not only helps to reduce stress and anxiety, but also the symptoms of depression that individuals may have. Therefore, the use of digital tools has shown a significant reduction in depressive symptoms. These facts are supported by numerous studies [35].

On the other hand, following the results obtained in the present study, it is evident that the use of digital technologies in interventions to improve the mental health of adolescents provides greater awareness and management of mental health. In this sense, various studies support the results such as those carried out by Chen et al. [36], where their findings indicated that digital interventions, including mobile applications, virtual reality and educational games, have a moderate but significant effect both in improving emotional well-being and in managing and raising awareness of mental health of adolescents. In addition, these tools have proven useful in addressing problems such as anxiety, depression and bullying. Therefore, other research, through literature reviews, analysed the effectiveness of digital interventions in the emotional regulation of adolescents. Some found that digital games were particularly effective in reducing negative emotional experiences in young people at risk of anxiety, although most studies are in early stages of development [37]. The effectiveness of self-directed digital interventions, such as mobile applications, has also been evaluated in adolescents aged 11 to 18 years, with the aim of determining their impact on emotional regulation, the reduction in psychopathology and the improvement of academic and social functioning [38].

Finally, the use of digital tools has proven to be a promising tool for improving the emotional well-being of children and adolescents, especially in the development of social skills and in strengthening emotional resilience. One study analysed the effectiveness of a digital intervention designed to improve emotional regulation and social communication skills in children with difficulties in these areas. After 14 weeks of intervention, participants demonstrated significant improvements in their ability to manage emotions and establish social relationships, with effects sustained up to six months later [39].

RQ2: What are the main challenges and barriers in the use of digital technologies for psychological well-being?

The analysis of the studies selected for the review revealed the main barriers and challenges in the use and employment of digital technologies for psychological well-being. Below are the main aspects that hinder their adoption:

- Accessibility: While digital applications and platforms can improve access to mental health, significant digital gaps persist. In particular, children and adolescents in low-income communities may lack access to electronic devices or a stable internet connection, which limits their participation in digital interventions [5]. This digital disparity reduces equity in access to mental health services and represents a significant obstacle in the implementation of technological solutions on a large scale [29].
- User Engagement: Despite their proven effectiveness, many of these digital tools experience low user retention. For example, the study by Shi et al. [24] on the Thought Spot platform showed that although young people perceive its functionality and usefulness as positive, most abandon the application within a short period of three weeks. This phenomenon is due, in part, to the lack of motivation and the design of the interventions, which do not always align with the preferences and needs of adolescents [26].
- Training and Acceptance by Professionals: The integration of digital tools into traditional models of psychological and psychiatric care requires that mental health professionals be trained in their use and trust in their effectiveness. However, the lack of specific training and resistance to change can hinder their adoption in clinical settings [17]. It is essential that digital technologies are accompanied by training programmes for mental health professionals, thus ensuring their proper implementation [8].
- Data Privacy and Security: The use of digital apps and platforms for mental health involves the collection and storage of highly sensitive data, raising ethical and legal concerns regarding privacy and security. One example is the Calm & Care app, which incorporated security mechanisms to ensure safe monitoring by parents (Nahreen Zannat & Mahmud, 2024). However, not all apps offer adequate security measures, which can lead to user mistrust and hinder widespread adoption [22].
- Scientific Evidence and Clinical Validation: Although studies indicate that digital interventions can reduce anxiety, stress and depression in children and adolescents, the lack of long-term clinical trials and qualitative studies limits the rigorous validation of these interventions [9]. For example, the Cuida tu Ánimo app demonstrated positive results in reducing depressive symptoms in adolescents, but its long-term effectiveness has not yet been confirmed with follow-up studies [25]. Standardisation in the design and evaluation of these tools is key to ensuring their applicability and reliability in clinical contexts [11].

Thus, we must bear in mind that despite the enormous potential of digital technologies to improve child and adolescent mental health, their adoption and long-term sustainability depend on addressing a number of challenges. Overcoming these barriers will require coordinated efforts between researchers, mental health professionals, and technology developers, in order to optimise the effectiveness and equity of these tools in clinical and educational practice.

RQ3: What guidelines can guide future research on the implementation of mental health technologies for children and young people?

Based on the results obtained, future research on the implementation of mental health technologies for children and young people should be guided by the following key aspects:

- Used as a complement to traditional therapy: Applications such as Cuida tu Ánimo have been shown to reduce depressive symptoms in adolescents [25]. These tools can be used as support between therapy sessions, offering practical exercises and educational resources that encourage treatment continuity and patient adherence.
- Remote monitoring and follow-up: Tools like Calm & Care allow parents and professionals to monitor the progress of young people, facilitating early intervention and personalization of treatment [22]. This is especially beneficial for managing fluctuating symptoms and optimising therapeutic strategies.
- Automation and efficiency for therapists: Gamification in mental health apps, as seen in tools such as Smartteen, incorporates game elements to improve user motivation and engagement. Studies such as Srivastava et al. [27] have shown that incorporating self-help and automated monitoring modules can effectively reduce depressive symptoms and optimise therapists’ time. These gamification elements, such as rewards and goals, encourage regularity and persistence in users. However, not all aspects of gamification are equally effective. For example, while rewards may increase participation in the short term, they may not sustain motivation in the long term without the support of more in-depth therapeutic interventions. In addition, scoring and competition systems may generate anxiety in some users, counteracting the potential benefits. It is crucial to identify and apply those gamification elements that align the objectives of the application with the specific psychological needs of the users, avoiding those that may introduce new stressors or diminish the effectiveness of the treatment.
- Education and awareness: Platforms like Thought Spot and POD Adventures have shown an increase in mental health awareness and stress management, being useful in school programmes [24,28]. Integrating these tools into educational curricula can strengthen mental health literacy and reduce the stigma associated with psychological disorders.
- Personalization of interventions: Gamification and interactive approaches, such as those used in Sokoon, can increase engagement and improve therapeutic outcomes [30]. The ability to tailor digital experiences to each user’s individual needs can improve adherence and motivation in treatment.
- Public health strategies: Governments and organisations can integrate these tools into mental health prevention and care campaigns in school, community, and clinical settings [10]. By providing access to validated digital resources, early detection of disorders can be improved and timely intervention facilitated.

Digital interventions can improve the accessibility and effectiveness of traditional models of psychological and psychiatric care if their challenges are addressed, ensuring an adapted, scientifically validated implementation aligned with the needs of users and mental health professionals. Combining digital approaches with traditional interventions can strengthen mental health care systems, facilitating equitable access and continuous improvement in treatments.

This finding reinforces the applicability of our results for mental health professionals, educators, and policymakers, highlighting the importance of integrating digital solutions into existing mental health frameworks to maximise their impact and effectiveness.

## 5. Conclusions

The findings of this systematic review show that digital interventions for child and adolescent mental health have a significant impact on reducing symptoms of anxiety, stress, and depression, as well as improving general well-being and mental health literacy. The inclusion of interactive tools, gamification, and remote monitoring strategies has been shown to improve user adherence and engagement, facilitating access to psychological interventions in various contexts. However, the effectiveness of these interventions largely depends on factors such as the quality of the design, integration with traditional mental health approaches, and parental involvement. Despite positive results, challenges in their implementation, such as acceptance by professionals, accessibility in vulnerable populations and data privacy, continue to be barriers that limit their widespread impact. The integration of these tools into health and education systems requires a multidisciplinary approach that considers their scientific validation, scalability and alignment with traditional psychological care models.

### 5.1. Limitations

While this study provides a comprehensive synthesis of the effectiveness of digital interventions in children’s mental health, it has some limitations that should be considered. First, the review focused only on studies published in English and in scientific articles, which could have excluded relevant research in other languages or formats.

Only quantitative studies have been included in this systematic review in order to objectively assess the effectiveness of digital interventions on child and adolescent mental health. This methodological decision is based on the need for measurable and comparable data to analyse the impact of these technologies in terms of symptom reduction, improved psychological well-being and other standardised indicators. Quantitative studies, in particular randomised controlled trials and longitudinal studies, offer greater methodological rigour and allow causal relationships to be established, which is essential to determine the real effectiveness of these interventions. Likewise, the exclusion of qualitative studies responds to the review’s objective of synthesising evidence based on objective and replicable metrics, facilitating the possibility of statistical analysis and meta-analysis. However, it is recognised that qualitative studies can provide valuable information on user experience and challenges in implementing these technologies, so future research could complement these findings with a mixed approach that considers both types of evidence. While the use of the PICO model to formulate the research question is appropriate and has allowed the study to be clearly structured, it is important to recognise that the reliance on quantitative studies may limit the analysis of subjective factors, such as user experience when interacting with digital mental health interventions. In this sense, future research could benefit from a mixed approach, incorporating qualitative methodologies such as in-depth interviews or case studies to better understand the perception, usability and adherence of these tools in children and adolescents.

Although the registration of systematic reviews on platforms such as PROSPERO is a recommended practice to improve transparency and prevent duplication of studies, in this case such registration was not performed because PROSPERO prioritises systematic reviews of clinical interventions and randomised controlled trials, while this review focuses on digital interventions without a direct clinical intervention. Nevertheless, a rigorous methodological approach has been followed, using PRISMA guidelines and quality assessment tools such as JBI and ROBINS-I, ensuring the transparency and reliability of the review process.

### 5.2. Future Lines of Research

Based on the findings and limitations identified, future research should focus on several key aspects. First, it is necessary to conduct longitudinal studies that allow the sustainability of the effects of digital interventions to be evaluated in the long term. Likewise, it is recommended to expand qualitative research to better understand the barriers and facilitators in the adoption of these tools by users and mental health professionals. In addition, it is essential to explore strategies to improve user retention and engagement, including the personalization of interventions and the use of artificial intelligence to adapt content to individual needs. Finally, further analysis is required on the implementation of these technologies in low-resource contexts, ensuring their accessibility and equity in access to mental health care.

Given that the studies analysed in this review focus on quantitative methodologies, future research should explore qualitative approaches to assess in depth the user experience and health professionals’ perception of these digital interventions. Furthermore, the application of the Cochrane model in future systematic reviews would improve the assessment of the quality of evidence and the consistency of the results, facilitating their integration into clinical and educational practice.

## Figures and Tables

**Table 1:** Information on included studies.

Author/Year	Methodological Design	Sample and Gender	Population and Age Group	Mental Health Condition(s)	Digital Intervention	Findings (Benefits and Limitations)	Country
Nahreen Zannat and Murni Mahmud, 2024 [22]	Quantitative	10 families. No gender is specified.	Children from 4 to 10 years old	Anxiety, stress and behavioural changes.	APP: Calm & Care	The app proved effective in addressing issues such as anxiety and stress stemming from the pandemic, promoting greater engagement and enthusiasm among children. It also facilitated active parental involvement through tools such as a dashboard to monitor progress and coordinate counselling sessions, all under strict privacy and security measures.	Bangladesh
Høgsdal, 2023 [23]	Quantitative	45 participants (25 girls and 17 boys)	Teenagers between 11 and 16 years old.	Stress and difficult emotions or situations	Applications: Opp and or NettOpp	The study indicates that young people are satisfied with both apps (Opp and NettOpp) to increase their knowledge about mental health and help them deal with stress and difficult situations. It also increases awareness about cyberbullying.	Norway Canada
Shi et al., 2021 [24]	Quantitative	131 participants (106 were female, 22 were male, and 3 identified themselves as non-binary).	Teenagers between 17 and 29 years old	Mental health and well-being support	Digital platform: Thought Spot	This platform helps transition-aged youth seek help from mental health and wellness resources. Participants highlighted its visual appeal, functionality, and usefulness; however, many stop using it within a short period of time (3 weeks).	Canada
Martínez et al., 2021 [25]	Quantitative	The study included 199 participants, of whom 106 were women and 93 were men.	Teenagers between 13 and 17 years old	Depression	Application: Take care of your spirit	Post-intervention results show a reduction in depressive and anxious symptoms in adolescents through digital interventions.	Chile
Osborn et al., 2020 [26]	Quantitative	103 students (gender not specified)	Teenagers between 13 and 18 years old	Stress, anxiety, depression..	Platform: Shamiri-Digital	The digital intervention produced a greater reduction in depressive symptoms in adolescents. Therefore, a brief digital and computer-based intervention may reduce depressive symptoms in adolescents in sub-Saharan Africa.	Kenya
Srivastava et al., 2020 [27]	Quantitative	21 adolescents (gender not specified)	Adolescents between 13 and 19 years	Depression	Web application: Smartteen	Smartteen is India’s first computer-assisted intervention for the treatment of depression in adolescents. Preliminary results suggest that it is feasible, acceptable and effective in reducing depressive symptoms and helps save therapist time.	India
Gonsalves et al., 2021 [28]	Quantitative	248 adolescents (124 males and 124 females).	Teenagers between 13 and 18 years old	Stress	Application: POD Adventures	Results showed improvements in mental health symptoms, stress and well-being. Participants found POD Adventures easy to use, engaging and helpful in solving their problems.	India
Rahayu et al., 2024 [29]	Quantitative	Not specified	Children between 13 and 18 years old	Anxiety and depression	Not specified	The use of mobile apps for adolescent mental health has great potential to improve access to and effectiveness of services. These apps can provide useful support, education, and interventions to help adolescents manage their mental health problems. Therefore, their implementation should be part of a comprehensive strategy to promote youth well-being.	Indonesia
Amer, N et al., 2023 [30]	Quantitative	5 participants (4 women and 1 man)	Young Arabs (no age range specified)	Depression, anxiety and stress	Sokoon	The tool proved to be practical, well-received and effective in reducing symptoms of anxiety and depression as it is based on cognitive behavioural therapy through gamification. In addition, it offers an accessible and culturally adapted alternative.	Egypt
Charvet et al., 2021 [31]	Quantitative	35 participants (28 women and 7 men).	Teenagers between 14 and 23 years old	Anxiety	Personal Zen	Was shown to be feasible and effective in reducing anxiety in paediatric patients with multiple sclerosis. Participants showed significant improvements in negative affect and anxiety following the intervention.	USA

## Supplementary Materials

The following supporting information can be downloaded at: https://www.mdpi.com/article/10.3390/children12030353/s1, Supplementary Materials S1 (Table S1: Checklist item), Supplementary Materials S2 (Table S2: Johanna Briggs Checklist (JBI)) and Supplementary Materials S3 (Table S3: Risk of bias ROBINS-I of the included studies; Figure S1: Distribution by year of publication).
